# Management of functional neurological disorder

**DOI:** 10.1007/s00415-020-09772-w

**Published:** 2020-03-19

**Authors:** Gabriela S. Gilmour, Glenn Nielsen, Tiago Teodoro, Mahinda Yogarajah, Jan Adriaan Coebergh, Michael D. Dilley, Davide Martino, Mark J. Edwards

**Affiliations:** 1 Department of Clinical Neurosciences, Faculty of Medicine, University of Calgary, Calgary, AB Canada; 2 Neuroscience Research Centre, Institute of Molecular and Clinical Sciences, St. George’s University of London, Cranmer Terrace, London, SW17 0RE UK; 3 Faculdade de Medicina, Instituto de Medicina Molecular, Universidade de Lisboa, Hospital de Santa Maria, Lisbon, Portugal; 4 Atkinson Morley Regional Neuroscience Centre, St. George’s University Hospitals NHS Foundation Trust, London, UK

**Keywords:** Functional neurological disorder, Functional movement disorder, Functional seizures, Psychogenic nonepileptic seizures, Psychogenic movement disorder

## Abstract

Functional neurological disorder (FND) is a common cause of persistent and disabling neurological symptoms. These symptoms are varied and include abnormal control of movement, episodes of altered awareness resembling epileptic seizures and abnormal sensation and are often comorbid with chronic pain, fatigue and cognitive symptoms. There is increasing evidence for the role of neurologists in both the assessment and management of FND. The aim of this review is to discuss strategies for the management of FND by focusing on the diagnostic discussion and general principles, as well as specific treatment strategies for various FND symptoms, highlighting the role of the neurologist and proposing a structure for an interdisciplinary FND service.

## Introduction

Functional neurological disorder (FND) is a common cause of persistent and disabling neurological symptoms [1]. These symptoms are varied and include abnormal control of movement (e.g. weakness, tremor, dystonic posturing), episodes of altered awareness resembling epileptic seizures (functional/dissociative seizures) and abnormal sensation. Fatigue, pain and cognitive difficulties are common additional symptoms. In recent years, there has been increasing emphasis placed on the role of neurologists in the management of FND. This replaces a traditional neurological approach of excluding a neurological disease process and either discharging the patient or referring to a psychiatrist. Instead, an extended role for neurologists can include a specific expertise in the diagnosis of FND, diagnostic explanation, treatment and follow-up, often acting as coordinator for a multidisciplinary team-based approach to management [2]. There may be comorbid psychiatric disorders present, and thus a coordinated effort between a neurologist and psychiatrist is important for both assessment and management on a case by case basis. It is, therefore, important for neurologists to understand the emerging evidence for various therapeutic options for this heterogenous group of patients. The purpose of this review is to discuss strategies for management of FND, highlighting the role of the neurologist, and proposing a structure for an interdisciplinary FND service.

## Diagnostic explanation and general principles of management

Management of FND, regardless of which specific symptoms are present, begins with a comprehensive assessment of the range of symptoms present, followed by explanation and discussion of the diagnosis [2]. People with FND are often polysymptomatic, and although it takes time, assessment of all the symptoms present is essential for an effective consultation and for planning future management. This process allows identification of symptoms beyond those of the “headline” functional symptom, including sleep disturbance, fatigue, pain, cognitive symptoms and co-morbid psychiatric symptoms, which can be important to address specifically in the management plan. These “non-motor” symptoms often have a greater impact than motor symptoms on health-related quality of life in FND patients [3]. In addition, it is common for patients with FND to have neurological, psychiatric and general medical comorbidities, which need to be acknowledged and incorporated as necessary in any management plan. Obvious mental health comorbidities can also initially be explored during this consultation and asking screening questions for anxiety and mood disorders after a thorough review of all other symptoms avoids the impression that FND symptoms are being attributed to their mental state alone. Asking about sleep and reasons for why people are on medication like selective serotonin reuptake inhibitors are often a good opening into exploring mental health. The process of asking about previous interactions with healthcare professionals, previous diagnostic explanations and what the patient believes to be the problem enables an appreciation of the often long journey patients have been on in reaching a diagnosis of FND and provides an understanding of previous interactions with healthcare professionals, previous diagnostic explanations and what the patient believes to be the diagnosis [2].

The diagnostic explanation should follow a “normal” structure similar to that used to deliver the diagnosis of other neurological disorders [2]. This would typically begin with naming the problem the patient has: “you have a functional neurological disorder”, and explaining this to be a common and genuine cause of neurological symptoms. As one would in any other disorder, it is useful to start with a mechanism or “how” level explanation. For FND this might use an analogy that explains that real and involuntary symptoms arise from malfunction rather than damage (e.g. software rather than hardware). We often use a disconnection analogy, explaining that the “basic wiring” of the nervous system is normal, as is the person’s will or motivation, but there is a disconnection between the two. This can in some situations be usefully demonstrated on physical examination; Hoover’s sign of functional weakness, and distractibility and entrainment in functional tremor are all usefully explained by a software malfunction or disconnection mechanism. Such demonstrations also reveal the potential reversibility of the condition [4].

Aetiological or “why” level discussions of the diagnosis may be important for some patients, but are best done from the standpoint of risk factors that can make some people more vulnerable to developing FND, rather than an obvious direct cause, and need not necessarily form part of the initial consultation. A systematic review has found that there is an association between childhood and adult stressful life events and maltreatment, particularly emotional neglect, and FND [5]. However, this review also highlighted that 14–77% of patients with FND do not report stressful life events, which in any case are also common in a general population without FND. In this way it is possible to discuss potentially important risk factors and maintaining factors, without suggesting that the patient has to have these in order for the diagnosis of FND to be correct. There are a number of online resources, which can be accessed by patients for additional information, such as https://www.neurosymptoms.org and https://www.fndhope.org. However, providing online resources alone, without further discussion and follow-up, is not usually sufficient, and instead can be perceived as dismissive by patients.

There are additional important management steps that can be taken during the first consultation, once the diagnosis of FND is made. It is prudent to develop a plan to stop medications that have been started without ongoing indication and may be causing harm, such as anti-epileptic medications in patients with functional seizures, opiates for chronic pain and psychotropic medications that have not been effective [6]. As the neurologist making the diagnosis, there is a unique opportunity to provide other healthcare providers with information about the diagnosis, thus helping to reduce the chance of future treatment with other potentially harmful medications or procedures. Basic education about distraction techniques during movement or sensory grounding before a functional seizure can be provided, as well as a discussion of graded exercise and pacing of activity for those with chronic pain and fatigue [2].

Functional symptoms are quite frequently comorbid with neurological, general medical or psychiatric illness, and thus concurrent sub-specialty management will occasionally be required. For example, a meta-analysis of frequency of dual diagnosis of epilepsy and functional seizures showed that among patients with epilepsy, the frequency of functional seizures was 12%, while among those with functional seizures, the frequency of epilepsy was 22% [7]. Co-morbid psychiatric disorders may require psychiatric intervention [5]. A difficult question that often arises is whether every patient diagnosed with FND should be evaluated by a psychiatrist. Ideally every patient would be seen by a psychiatrist and/or a psychologist as part of an integrated approach where they are part of the team rather than in a separate organisation, to aid in assessment for psychopathology or the sequelae of stressful life events. However, this is often not available or always practical, and thus it is necessary to create a robust triage system for appropriate referrals within a multidisciplinary FND clinic. This system, which could use questionnaire-based assessment of psychopathology and/or specific triage questions, could usefully be developed with local psychiatric/neuropsychiatric expertise. This would allow for the appropriate ongoing referral for expert psychiatric assessment and treatment for those patients who in particular have high levels of complexity, diagnostic uncertainty regarding psychiatric comorbidity, treatment-resistant psychiatric illness or high levels of risk including deliberate self-harm and suicide risk. Screening measures for mood include the Patient Health Questionnaire (PHQ-9), and for mood and anxiety, the Hospital Anxiety and Depression Scale (HADS) and General Anxiety Disorder (GAD-7) measures [8–10]. Where a straightforward presentation of depression or anxiety is identified, it is often appropriate for the neurologist or primary care physician to treat these comorbidities according to established guidelines, with referral to psychiatry if required [11, 12].

Diagnostic explanation and these basic management steps can often be an effective treatment. They can also prevent unnecessary and potentially harmful further investigations and treatments. A systematic review exploring the impact of receiving a diagnosis of functional seizures found that approximately half of patients had a reduction or cessation of attacks, although considerable heterogeneity exists between studies [13]. However, diagnostic explanation alone frequently does not yield resolution of symptoms. In a systematic review assessing the prognosis of functional motor symptoms, a majority of included studies (20 of 24) found that greater than one-third of patients had ongoing symptoms at follow-up, with the severity being the same or worse [14]. Thus, for many people with FND it is necessary to consider symptom-specific management strategies and to arrange follow-up, which allows for the monitoring of symptoms, the ability to review old notes and test results and the opportunity for further diagnostic explanation and discussion.

## Functional motor symptoms

There is considerable heterogeneity amongst patients who present with motor symptoms, which may include weakness, gait disorders and movement disorders such as tremor, dystonia, fixed postures, jerks and tics. It is, therefore, necessary to tailor individual therapy to the patient and their symptoms, considering symptom type, comorbidity and acceptability/personal preference. Treatments for motor symptoms described in the literature include specialist physiotherapy, multidisciplinary rehabilitation, specialist cognitive behavioural therapy or psychotherapy; as well as novel interventions or treatment adjuncts such as transcranial magnetic simulation (TMS), botulinum toxin, therapeutic sedation, hypnosis and electromyographic biofeedback [15]. There is no “one-size-fits-all” therapy available, and thus careful patient selection and individualized therapy is required. As an example of the importance of patient selection, in a feasibility study of a 5-day outpatient physiotherapy program, 210 patients were assessed for eligibility, with only 60 (29%) deemed suitable [16]. Patients were most commonly excluded due to excessive pain or fatigue, or co-existing psychological symptoms requiring treatment [16].

Physical-based therapies have the most robust evidence for functional motor symptoms, with the rationale being that abnormal movement patterns that develop outside of a patient’s control, coupled with a heightened level of self-directed attention, can be retrained [17]. The goal is to intervene on a patient’s illness belief and show that distraction away from the abnormal movements can temporarily resolve symptoms, such as can be demonstrated when distracting a functional tremor [15]. Thus, physiotherapists, occupational therapists, speech therapists, clinical psychologists and other similar clinicians have an important role in providing further education around illness beliefs and self-directed attention, demonstrating that normal movement is possible, retraining movement by diverting attention away from functional symptoms and changing maladaptive behaviours [15].

In terms of delivery of rehabilitation, various inpatient and outpatient programs have been developed and studied. An intensive 5-day outpatient physiotherapy protocol showed a good outcome in 70% of patients at 6 months following treatment [16]. Multiple studies assessing multidisciplinary inpatient rehabilitation, varying in duration from 3 to 14 weeks and combining neuropsychiatry, psychology, physiotherapy, occupational therapy and occasionally speech therapy, have shown that the majority of patients had a significant improvement in physical function and quality of life [18–20]. In terms of length of treatment, there is a recent retrospective cohort study from a hospital-based outpatient physical therapy program that demonstrated a statistically significant relationship between the number of sessions attended by patients and clinical improvement [21]. It is not clear at this time if there is a superior therapy program, but with appropriate patient selection, what is clear is that the most valuable and important aspect of therapy is the specificity of treatment to FND. The ideal setting for treatment is unknown and likely is affected by complexity, severity and chronicity of symptoms [15]. Inpatient programs allow for higher intensity treatment, while limiting environmental and social factors that may be perpetuating symptoms. Meanwhile, the outpatient setting can allow for delivery of treatment over a longer duration in an environment that is more similar to a patient’s home environment, perhaps reducing the risk of relapse at discharge.

## Functional seizures

The gold standard for diagnosis of functional seizures is video-electroencephalography. However, there are circumstances where this is not necessary (based on the presence of particular clinical characteristics) or because it is not feasible (e.g. in people with seziures only occasionally). After diagnosis, a discussion around symptoms and experiences at the onset of the attack can be very helpful. Many patients are able to identify warning symptoms, which frequently lead up to an attack. The ability of a patient to recognize these symptoms may allow them to practice sensory grounding techniques, where they deliberately focus their attention elsewhere in order to prevent the attack from occurring, thus leading to a reduction in attack frequency [22, 23].

The most robust evidence for treatment of functional seizures, though largely from non-randomised, uncontrolled studies, is for cognitive behavioural therapy (CBT) delivered by appropriately trained mental health professionals. A number of CBT approaches have been used, with the two most clearly described models being one which focuses on the cognitive, emotional, physiologic and behavioural aspects of functional seizures, and one which views functional seizures as a response to distressing cues, utilizing a fear escape-avoidance model [24]. A systematic review found that non-randomized studies showed there to be benefits of CBT for functional seizures, with one randomized clinical trial reporting a significant reduction in non-epileptic attack frequency and improved quality of life at the end of treatment, with a trend to benefit at 6 months [25]. A recent consensus statement following systematic review recommends CBT as first-line therapy for adults with functional seizures [6]. A pilot study has also shown that CBT-based group psychoeducation is an effective treatment for decreasing attack frequency and improving overall wellbeing [26].

In terms of pharmacologic therapy, there are no specific medications shown to be effective in treating functional seizures [6]. Instead, it is recommended to assess for and treat co-existing psychiatric disorders on an individual basis, as well as to discontinue antiepileptic therapy in patients without comorbid epilepsy [6]. Specifically, post-traumatic stress disorder, major depressive disorder and anxiety disorders have been reported at high rates in patients with functional seizures and should be treated if present [27, 28]. One randomized clinical trial demonstrated a significant reduction of functional seizure frequency for patients treated with both CBT and sertraline (59%), as well as with CBT alone (51%), while sertraline alone did not significantly reduce seizure frequency (27%) [29]. Worth noting is that the CBT only group reported a greater improvement of secondary outcomes (quality of life, depression, anxiety, somatic symptoms, impulsivity and psychosocial functioning) as compared to CBT plus sertraline, perhaps owing to medication adverse effects in a somatically-focused population of patients.

## Functional cognitive disorders

Functional cognitive disorders encompass a group of memory-related symptoms that are distressing and cause impairment in day-to-day functioning, with inconsistency present between self-reported symptoms and neuropsychological testing [30]. Patients presenting to memory clinics, found to have no evidence of underlying neurodegenerative or neurological cause for their symptoms, encompass a diverse group with varying aetiologies for their symptoms [31]. A number of common and overlapping clinical profiles have been identified, including: (1) memory symptoms related to depression or anxiety, (2) ‘normal’ memory lapses that become the focus of attention, (3) health anxiety about dementia, (4) functional memory disorder, (5) memory symptoms as part of another functional disorder, (6) retrograde dissociative amnesia, (7) memory symptoms secondary to medication or drug use, (8) disease other than dementia causing memory symptoms, (9) functional memory symptoms in patients who later go on to develop dementia or another neurologic disease and (10) exaggeration or malingering [31]. Due to this heterogeneity, it is necessary to carefully assess a patient presenting with memory complaints suspected to be functional, specifically attempting to identify an underlying cause such as medical conditions (i.e. chronic obstructive pulmonary disorder), sleep apnoea, primary insomnia, anxiety disorders, major depressive disorder, or medication and substance use.

Little evidence exists to guide specific treatment strategies for people with functional cognitive disorders. General treatment strategies, as described earlier, are very important. It is particularly important avoid or reduce medications that may worsen memory symptoms [31]. It is also helpful to normalize the experience of forgetting. CBT focused on challenging unhelpful thoughts and patterns of avoidance of memory use may be helpful [31]. Group therapy involving education and stress management was shown to be effective in improving self-rated memory at 6-month follow-up [32].

## Functional sensory symptoms

A number of functional sensory symptoms may be present, including visual symptoms, somatosensory symptoms, including paresthesia and anaesthesia, and functional hearing loss or tinnitus. These frequently occur alongside other functional symptoms [33]. There is very little evidence on specific treatment strategies for these conditions, and thus it is recommended to apply general principles of management and provide specific treatment for comorbid symptoms. There is a report in two patients with functional visual loss of the successful use of occipital TMS to produce flashes of light in patients’ visual fields, as well as for supplemental hypnotherapy [34]. Large-field repetitive TMS over the centro-parietal area has been described for treatment of functional somatosensory symptoms in a case series of 12 patients, with a complete recovery observed in 6 patients and partial recovery in 3 patients [35]. There is a significant need for the investigation of management strategies for functional sensory symptoms.

## Other functional symptoms

There is a wide array of other neurologic symptoms affecting patients, including functional urinary symptoms, persistent postural perceptual dizziness (PPPD), functional speech and voice disorders (FSVD), functional dysphagia and globus sensation. Treatment approaches vary based on symptoms, with varying quality of evidence available [36].

Fowler’s syndrome, commonly seen in young women, has been classically described as urinary retention due to impaired urethral sphincter relaxation, impaired sensation of bladder fullness, and is often triggered by events such as surgery, childbirth or minor medical procedures [37]. It has frequently been found to be associated with FND, chronic pain and psychological symptoms [37]. If Fowler’s syndrome is suspected, referral to neuro-urology services (if available) should be considered, as treatments including sacral neuromodulation have been shown to be effective [38].

PPPD is a functional disorder in which patients experience dizziness and unsteadiness, worse when upright, moving and in settings with complex visual stimuli [39]. Vestibular therapy targeted at PPPD specifically, which aims to gently and slowly habituate the abnormal responses to movement and visual stimuli, has been shown to be effective. An experienced vestibular therapist may aid patients through habituation exercises incorporating CBT principles, thus allowing a gradual return to normal functioning [39]. There is emerging evidence for CBT, as well as selective serotonin reuptake inhibitors and serotonin norepinephrine reuptake inhibitors, even in the absence of overt psychopathology [40]. In some cases, patients may experience a functional gait disorder secondary to PPPD, which may be amenable to physical-based therapies, as described above.

FSVD frequently occurs alongside other functional symptoms, for example occurring at a rate of 16.5% in a cohort of patients with functional movement disorders [41]. Patients frequently experience stuttering, speech arrests, foreign accent syndrome, hypophonia and dysphonia [41]. A multidisciplinary approach including intensive voice therapy provided by a specialised speech therapist, symptomatic vocal exercises and management of any comorbidities is recommended, although only poor quality evidence exists [42].

Patients presenting with dysphagia and globus sensation frequently require additional investigations with otorhinolaryngology to rule out structural causes before a diagnosis of functional dysphagia may be given. Again, evidence for treatment is limited, but typically includes education around the diagnosis, avoidance of precipitating foods, adequate mastication and trial of proton-pump inhibitor [43]. There is also a potential role for speech therapy for patients experiencing globus sensation [44].

## Comorbid pain, fatigue and “cognitive fog”

There is a high frequency of pain, including chronic migraine, fatigue and the subjective sense of “cognitive fog” in patients with FND [45–47]. Although distinct entities, there appears to be significant overlap with FND, fibromyalgia (FM) and chronic fatigue syndrome (CFS), particularly in the way patients describe subjective cognitive difficulties, perhaps underpinned by excessive attention towards the body, severe pain and fatigue [48]. It is necessary to recognize and treat these symptoms as part of FND, as they may be acting as either triggering or maintaining factors in FND. No strong evidence exists for the management of fatigue and chronic pain in FND, but a potential therapeutic approach would be to address these symptoms in a similar fashion to CFS and FM, respectively, with the caveat that these diagnoses likely encompass a range of different aetiologies.

CFS is a heterogenous disorder with disabling fatigue, as well as chronic pain, headaches, sleep disturbances, autonomic/neuroendocrine/immune manifestations and cognitive fog [49–52]. A multidisciplinary approach to therapy has been proposed, including CBT, physiotherapy, exercise therapy and pacing strategies [49–52]. To date, there are no recommended pharmacological treatments for CFS [52].

FM is characterized by widespread pain, along with multiple other symptoms including fatigue and cognitive fog [53–57]. As in CFS, a multidisciplinary approach to management is recommended, often accessed through chronic pain clinics. This approach may include non-pharmacologic management including education around pacing, CBT and exercise [53, 54].

Although the pathophysiology of FM is not completely understood, there is evidence that FM may have a neuropathic pain component, and there may be a deficit in central nervous system inhibition [58, 59]. Treatments aimed at modulating cortical excitability, both pharmacologic and non-invasive brain stimulation, appear to be effective in symptom improvement. A number of pharmacologic treatments have been studied. A meta-analysis demonstrated a significant improvement in pain intensity with the use of pregabalin for a small proportion of patients (10%) [55]. Pregabalin exerts its therapeutic effect by modulating cortical excitability, increasing the cortical silent period and the short intracortical inhibition [60]. Meanwhile, a meta-analysis of seven randomized, double-blind, placebo-controlled trials investigating the use of duloxetine for FM found it to be more effective than placebo at improving pain, but with considerable risk of side effects [57]. The analgesic effect of duloxetine may be linked to changes in frontal quantitative EEG [61]. The combination of pregabalin and duloxetine has been shown to be more effective than pregabalin monotherapy [62]. Only very low-quality evidence for the use of gabapentin in FM exists, and thus there is uncertainty about the risk–benefit ratio [56].

Non-invasive brain stimulation, including transcranial direct current stimulation (tDCS) and TMS, has been shown to be potentially of use in the treatment of FM, with the primary motor cortex and dorsolateral prefrontal cortex being the most common stimulation sites in FM [63]. Specifically, primary motor cortex stimulation may be better at reducing pain, while DLPFC stimulation may be better for fatigue and comorbid depression [63]. Both tDCS and TMS may be feasible and safe adjuncts to the treatment of FM; however, evidence remains too limited to guide optimal stimulation parameters [63]. Recently, it has been shown that peripheral transcutaneous electric nerve stimulation of pain inhibitory pathways reduced movement-evoked pain and fatigue [64]. Ultimately, the management of chronic pain and fatigue in FND is difficult. There is currently no evidence for the use of non-invasive brain stimulation for the treatment of chronic pain and fatigue in patients with FND, without a diagnosis of FM. However, it may be possible to use insights from trials in FM to develop sham-controlled trials of new clinical interventions for FND.

Thoughtful consideration should be made to the treatment of coexisting chronic headache. There is one study supporting the notion that neurological co-morbidity in FND might be undertreated. Elliott et al. describe that 23 of 43 (53.5%) functional seizure patients versus 22 of 29 (75.9%) epilepsy patients felt their migraines were adequately treated (*p* = 0.054). Furthermore, 19 of 43 (44.2%) patients with functional seizures and headache had never been prescribed a medication to abort migraines nor any prophylactic medication [65].

## Managing chronic disability

Despite a good diagnostic explanation and multidisciplinary therapy, some patients will be refractory to treatment, resulting in chronic disability. If a treatment plan is failing to provide benefit to a patient, it is reasonable to discontinue this approach. In this difficult situation, it is necessary to have a frank discussion with patients about the rationale for discontinuing active therapy, emphasizing that this may allow patients more time to spend on meaningful activities. Neurologists can provide patients with support in other ways, advocating for their access to social services, mobility aids and environmental adaptations, thus promoting quality of life, while ensuring patient safety. Physiotherapists may equip patients with appropriate mobility aids, and occupational therapists may aid in home adaptations [2, 15].

## Conclusion and setting up FND care pathways

FND is a heterogenous disorder, which requires careful assessment, open and holistic discussion of diagnosis and individualized therapy. As evidence emerges for various treatment options, it becomes more necessary to develop a strategy to allow patients to appropriately access the healthcare system. A stepped care model has been proposed in Scotland, outlining when patients may be managed by general practitioners and when patients should be referred to general neurology, specialized therapists or subspecialist neurologists, thus allowing for the most appropriate usage of limited resources [66]. Currently, specialist FND clinics are rare in most healthcare systems, but hopefully will become more common, improving access for complex patients requiring interdisciplinary care.

**Fig. 1:**
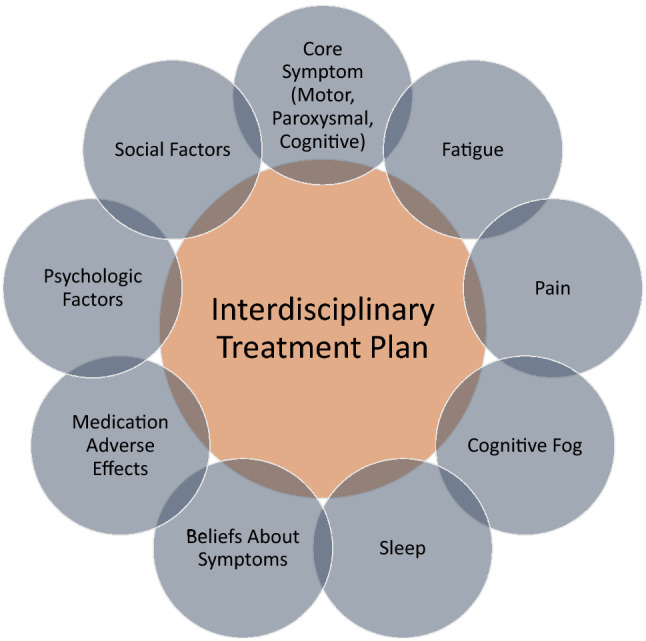
Interdisciplinary FND treatment framework emphasizing a holistic approach to the care of FND patients

Evidence for how best to structure an FND clinic is lacking in the literature, but it seems reasonable to consider a model similar to that of other complex-care clinics, emphasizing an interdisciplinary approach, with each involved discipline offering their own expertise in patient care (Fig. 1). Necessary elements include a thorough initial assessment, a robust triage system for referral to appropriate treatment (physical therapies, psychiatry, clinical psychology, day patient or inpatient rehabilitation) and follow-up. In one example of a clinic structure for functional movement disorders, patients attend a half-day clinic where they are assessed by a movement disorders specialist, psychologist, physical therapist and social worker, who then together discuss a plan for treatment with the patient [67]. Such a service can only work, however, if general neurologists, primary care physicians and community rehabilitation services continue to manage and follow a proportion of people with FND, in particular those with milder symptoms and those with chronic symptoms despite treatment, who are unlikely to improve in the long term.

Most patients will likely attend an FND clinic via general neurology, but a structure should be in place for earlier referral from acute services, including the emergency department and inpatient stroke or neurology services. This draws attention to the fact that all neurologists play an important role in the care of FND patients. Ultimately, it must be recognized that for many patients, FND is a chronic condition with multiple contributing factors, and thus ongoing support and follow-up will often be necessary. Relapses or exacerbation of symptoms frequently occur, and thus patients must also be supported to manage this to the extent that is possible, on their own, highlighting the importance of developing a self-management plan. It is also important to acknowledge that if new persistent symptoms emerge, these need careful assessment and potentially investigation in a normal manner, despite the diagnosis of FND.

One approach to encouraging self-managed care is to develop a workbook with patients that they may later refer to, which provides (1) an explanation of the patient’s problems, (2) discussion of triggering factors, (3) reflection on effective treatments that have been delivered including strategies that helped normalize symptoms, (4) markers of progress, (5) future goals and (6) plans to manage setbacks [15]. Ideally, following treatment and with a robust self-management plan in place, most patients will be equipped to be discharged from regular follow up in specialist FND services, but with a clear and rapid route back in if new symptoms develop or other help is needed. Improving knowledge and skills amongst community therapy and primary care teams is an important part of this aspiration.
